# Complement component 3 and complement factor H protein levels are altered in brain tissues from people with human immunodeficiency virus: A pilot study

**DOI:** 10.3389/fnagi.2022.981937

**Published:** 2022-08-31

**Authors:** Jerel Adam Fields, Mary Swinton, Erin E. Sundermann, Nicholas Scrivens, Kaylie-Anna Juliette Vallee, David J. Moore

**Affiliations:** Department of Psychiatry, University of California, San Diego, San Diego, CA, United States

**Keywords:** complement factor H, complement—immunological term, Alzheimer’s disease, HIV, beta—amyloid peptide, complement component 3

## Abstract

People with HIV (PWH) continue to suffer from dysfunction of the central nervous system, as evidenced by HIV-associated neurocognitive disorder (HAND), despite antiretroviral therapy and suppressed viral loads. As PWH live longer they may also be at risk of age-related neurodegenerative diseases such Alzheimer’s disease (AD) and its precursor, amnestic mild cognitive impairment (aMCI). The complement system is associated with deposition of AD-related proteins such as beta amyloid (Aβ), neuroinflammation, and neurological dysfunction in PWH. Complement component 3 (C3) is a key protagonist in the complement cascade and complement factor H (CFH) is an antagonist of C3 activity. We investigated the relationship between C3 and CFH levels in the brain and Aβ plaques and neurological dysfunction in 22 PWH. We analyzed by immunoblot C3 and CFH protein levels in frontal cortex (FC) and cerebellum (CB) brain specimens from PWH previously characterized for Aβ plaque deposition. C3 and CFH protein levels were then correlated with specific cognitive domains. C3 protein levels in the FC were significantly increased in brains with Aβ plaques and in brains with HAND compared to controls. In the CB, C3 levels trended higher in brains with Aβ plaques. Overall C3 protein levels were significantly higher in the FC compared to the CB, but the opposite was true for CFH, having significantly higher levels of CFH protein in the CB compared to the FC. However, only CFH in the FC showed significant correlations with specific domains, executive function and motor performance. These findings corroborate previous results showing that complement system proteins are associated with HAND and AD neuropathogenesis.

## Introduction

Central nervous system dysfunction remains a major problem for people with HIV (PWH) even in the presence of antiretroviral therapy (ART) (Cysique et al., 2021). HIV-associated neurocognitive disorders (HAND) persist in approximately 43% of PWH (Heaton et al., 2011; Wang et al., 2020). The developments in ART have led to increased longevity among PWH so that the population of PWH in the United States is now predominantly over the age of 50. However, this translates to increased susceptibility to age-related neuropathogenesis such as Alzheimer’s disease (AD) and its precursor, amnestic mild cognitive impairment (aMCI) (Althoff et al., 2022). Indeed, multiple studies from independent labs have implicated age-related proteinopathies in neuropathogenesis in PWH (Achim et al., 2009; Gisslen et al., 2009, 2019; Chen et al., 2014; Fields et al., 2018, 2020; Murray et al., 2022). Consequently, there is a need to better understand mechanisms of age-related neuropathogenesis in PWH.

Interest in the role of innate immune function in the brain has mounted as multiple studies have implicated a prolonged innate immune response as a potential etiology of neurodegenerative diseases (Heneka et al., 2014, 2015; Kim et al., 2021). The complement system is integral to innate immunity and it can be activated early on during immune responses (Merle et al., 2015a,b; Morgan, 2015). When controlled, the complement system plays a protective and even developmental role in the brain. However, there is ample evidence that, when dysregulated, the complement system may contribute to the pathogenesis of neurodegenerative diseases (Liddelow et al., 2017) such as HAND (Bryant et al., 2016; Nitkiewicz et al., 2017) and AD (Goetzl et al., 2018; Morgan, 2018).

Complement component 3 (C3) is a central player in the activation of both the classical and alternative activation pathways (Merle et al., 2015a,b). Complement factor H (CFH) acts as a negative regulator of C3, blocking the activation of the complement system on targeted cells that are meant to be protected from complement activity (Merle et al., 2015a,b). Recent studies have suggested that either the blocking of C3 or the augmentation of CFH may serve as a therapeutic strategy in neurodegenerative diseases (Li et al., 2012; Lukiw and Alexandrov, 2012; Lukiw et al., 2012; Hoh Kam et al., 2013). However, other studies show that C3 mediates clearance of Aβ plaques (Wyss-Coray et al., 2002; Maier et al., 2008). Clinically, low CSF C3 and CFH has been associated with worsening cognitive decline in MCI (Toledo et al., 2014; Hu et al., 2016). CFH genetic variants as well as expression of miRNA that target expression of CFH have been associated with increased risk for AD (Li et al., 2012; Lukiw and Alexandrov, 2012; Lukiw et al., 2012; Zhang et al., 2016). Thus the interplay between C3 and CFH may contribute to the neuropathogenesis of aging and age-related diseases in PWH.

Researchers from our group previously examined associations between plasma inflammatory biomarkers (e.g., C3, cystatin C, interleukin 6, soluble CD163, and soluble CD14) and metabolic syndrome (MetS) in 79 virally suppressed, older PWH compared to 47 human immunodeficiency virus (HIV)-seronegative controls (Bryant et al., 2016). Among PWH, higher C3 levels were significantly associated with MetS in addition to the individual MetS components of obesity, type II diabetes, dyslipidemia and hypertension. C3 levels were significantly higher in PWH with MetS vs. PWH without MetS, whereas no associations between C3 levels and MetS were observed among HIV-seronegative controls. Other studies show that HIV induces C3 expression in the brain (Nitkiewicz et al., 2017). Furthermore, C3 expression is associated with mitochondrial dysfunction in AD (Sekar et al., 2015), representing another link to the neuropathogenesis of AD. These findings suggest that C3 may be a marker of inflammatory processes that contribute to metabolic risk and, as examined in this proposal, brain function and AD risk. This may be particularly important because mitochondrial dysfunction is implicated in HAND and AD neuropathogenesis.

Aβ deposition is altered in the brains of PWH and may be associated with premature aging in this population. HIV infection of the brain is associated with increased levels of Aβ (Giometto et al., 1997; Esiri et al., 1998; Green et al., 2005), including intraneuronal Aβ (Achim et al., 2009) and other AD-related biomarkers including increased phospho-tau (ptau) (Brew et al., 2005; Clifford et al., 2009), and inflammatory cytokines, such as tumor necrosis factor (TNF)-α and interleukin (IL)-1β (Ortega and Ances, 2014; Gelman, 2015; Levine et al., 2016; Fields et al., 2018). In a study of frontal cortex (FC) samples from PWH, Aβ plaques were detected in 29% of 279 cases (Umlauf et al., 2019). To date, numerous studies have provided evidence of AD-related neuropathogenesis in HIV (Chen et al., 2014; Fields et al., 2018; Gisslen et al., 2019; Chemparthy et al., 2021; Murray et al., 2022), and thus, there is a need to better understand the common neuropathogenic mechanisms in AD and HAND.

In this study we utilized the post-mortem tissue repository, National NeuroAIDS Tissue Consortium (NNTC), to investigate C3 and CFH levels in the FC and cerebellum in older HIV + cases previously characterized for AD-associated neuropathology. We next examined how C3 and CFH levels relate to aMCI and HAND classification and performance on specific cognitive domains based on an antemortem cognitive evaluation within 18 months of death.

## Materials and methods

### Study population

For the present study, we evaluated brain tissues from a total of 22 older (at-least 50 years of age at death) HIV + donors from the NNTC [Institutional Review Board (IRB) #080323]. All studies adhered to the ethical guidelines of the National Institutes of Health and the University of California, San Diego. These cases had neuromedical and neuropsychological examinations within a median of 12 months before death. Subjects were excluded if they had a history of CNS opportunistic infections or non-HIV-related developmental, neurologic, psychiatric, or metabolic conditions that might affect CNS functioning (e.g., loss of consciousness exceeding 30 min, psychosis, etc.).

### Neuromedical and neuropsychological testing

Participants underwent a comprehensive neuromedical evaluation that included assessment of medical history, structured medical and neurological examinations, and the collection of blood, cerebrospinal fluid (CSF), and urine samples, as previously described (Woods et al., 2004; Heaton et al., 2010). Clinical data [plasma viral load (VL), postmortem interval, CD4 count] were collected for the donor cohorts. A neuropsychological battery assessed seven cognitive domains commonly affected by HIV: verbal fluency, working memory, processing speed, episodic memory for verbal and visual stimuli, executive function, and complex motor function. Specific tests are described elsewhere (Cysique et al., 2011). Raw test scores were transformed into demographically adjusted T-scores, including adjustments for age, education, gender and race, based on normative samples of HIV-participants (Norman et al., 2011). As part of the neuropsychological battery, participants also completed self-report questionnaires of everyday functioning (i.e., Lawton and Brody Activities of Daily Living questionnaire (Lawton and Brody, 1969), and/or Patient’s Assessment of Own Functioning; PAOFI (Chelune and Baer, 1986; Chelune et al., 1986).

### HIV-associated neurocognitive disorder classification

Participant’s performance on the neuropsychological test battery and their responses to the everyday functioning questionnaires were utilized to assign HAND diagnoses following Frascati criteria (Antinori et al., 2007). A HAND diagnosis required impairment in at-least two cognitive domains, defined by performance of at-least 1.0 standard deviation (*SD*) below the demographically adjusted normative mean on neuropsychological tests. HAND status was further categorized as asymptomatic neurocognitive impairment (ANI; no interference in everyday function), mild neurocognitive disorder (MND; at-least mild interference in everyday function), and HIV-associated dementia (HAD; marked interference in everyday function).

### Amnestic mild cognitive impairment classification

aMCI was classified using a version of the established, neuropsychological Jak/Bondi criteria (Bondi et al., 2014) that was previously adapted for the use of detecting aMCI symptoms amid a background of HAND in PWH (Sundermann et al., 2021). The Jak/Bondi criteria for aMCI requires two impaired tests (i.e., >1 *SD* below demographically corrected mean) within the memory domain. In order to capitalize on the retention deficit that is unique to aMCI/AD rather than the retrieval deficit that is common to both aMCI/AD and HAND, the Jak/Bondi MCI criteria was adapted to require at least one of the two impaired memory tests be a recognition test. The memory outcomes used in these criteria were the demographically adjusted T-scores of the Hopkins Verbal Learning Test-Revised (HVLT-R) and the Brief Visuospatial Memory Test-Revised (BVMT-R) (Norman et al., 2011) delayed recall and recognition subtests. Participants were classified as aMCI + if they showed impaired performance (T-score < 40) on at-least two of the four measures with at-least one of the impaired scores being a recognition measure. Of important note, the HAND classification criteria used in this study included BVMT-R and HVLT-R learning and delayed recall, but not recognition, scores to assess the learning and memory domain.

### Immunoblot analysis of complement proteins

Further assessment of the expression levels of C3 and CFH in NNTC cases were performed by immunoblot analysis. Cerebellum and FC was homogenized in buffer (1.0 mmol/L HEPES (Gibco, cat. no. 15630–080), 5.0 mmol/L benzamidine, 2.0 mmol/L 2-mercaptoethanol (Gibco, cat. no. 21985), 3.0 mmol/L EDTA (Omni pur, cat. no. 4005), 0.5 mmol/L magnesium sulfate, 0.05% sodium azide; final pH 8.8) as described in a previous publication (Swinton et al., 2021). In brief, as previously described (Fields et al., 2013), tissues from brain samples (0.1 g) were homogenized in 0.7 ml of fractionation buffer containing phosphatase and protease inhibitor cocktails (Calbiochem, cat. nos. 524624 and 539131). Samples were precleared by centrifugation at 5000 × g for 5 min at room temperature. Supernatants were retained as the whole lysate and stored at –80 until use. As previously described (Swinton et al., 2021), after determination of the protein content of all samples by bicinchoninic acid assay (Thermo Fisher Scientific, cat. no. 23225) and denaturing in lamellae sample buffer (Bio Rad, cat. no. 1610747), whole lysates were loaded (15 μg total protein/lane) on 4–15% Criterion TGX stain free gels (Bio Rad, cat. no. 5678085) and electrophoresed in Tris/Glycine/SDS running buffer (Bio Rad, cat. no. 161–0772) and transferred onto LF PVDF membrane with Bio Rad transfer stacks and transfer buffer (Bio Rad, cat. no 1704275) using Bio Rad Trans Blot Turbo transfer system. After the transfer, total protein was imaged using Bio Rad ChemiDoc imager under the stain free blot setting for normalization purposes. Total protein transferred to the blot membrane, rather than the traditional use of housekeeping genes such as beta actin and glyceraldehyde phosphate dehydrogenase, was used to normalize protein expression because studies show that “housekeeping” genes are often altered in processes associated with neurodegenerative diseases (Kish et al., 1998; Mahmood et al., 2021). Therefore, our results are presented as the expression levels of the target gene compared total protein transferred to the blot membrane, a more accurate representation of protein expression in any given lysate. The membranes were then blocked in 1% casein in tris-buffered saline (TBS) (Bio Rad, cat. no. 1610782) for 1 h. Membranes were incubated overnight at 4°C with primary antibodies diluted in blocking buffer. All blots were then washed in PBST, and then incubated with species-specific IgG conjugated to HRP (American Qualex, cat. no. A102P5) diluted 1:5,000 in PBST and visualized with SuperSignal West Femto Maximum Sensitivity Substrate (ThermoFisher Scientific, cat. no. 34096). Images were obtained, and semi-quantitative analysis was performed with the ChemiDoc gel imaging system and Quantity One software (Bio-Rad).

### Statistical analysis

We first examined and removed outliers in C3 and CFH protein levels as defined by levels greater than 3 SD from the sample mean. Statistical analysis was conducted using student *t*-test, one-way ANOVA, and effect size. Error bars represent standard error of the mean. Significance was set at a threshold of *p* < 0.05. We conducted a series of one-way ANOVAs to test mean differences in C3 and CFH protein expression levels in the FC and cerebellum by HAND classification and by aMCI classification. If there was a significant difference by HAND status, then we probed this difference by comparing protein expression levels among individual HAND types. We determined effect size of mean differences using Cohen’s *d*. We conducted a series of Spearman’s rank-order correlation, the non-parametric version of the Pearson product-moment correlation, to examine relationships between C3 and CFH expression levels in the FC and cerebellum and cognitive domain t-scores.

## Results

One outlier for frontal and cerebellar CFH protein levels was identified and removed from analyses resulting in a final sample of *N* = 22 for C3 and *N* = 21 for CFH protein levels in the FC and *N* = 14 for C3 and *N* = 13 for CFH in the cerebellum. See Table 1 for sample characteristics of the largest sample (*N* = 22) by HAND and aMCI classification. Overall, age at death ranged from 50 to 66 (mean = 56.2, *SD* = 4.6) and the sample was 82% male and 73% Caucasian. Fifteen (68%) of the participants were classified as HAND with the following break-down: 7 (32%) ANI, 3 (14%) MND and 5 HAD (23%). Ten participants (45.5%) were classified as aMCI, with only one of these cases classified as non-HAND, suggesting that this case only exhibited memory impairment.

**TABLE 1:** Clinical characteristics of HIV + brain tissues.

Variables	Cognitively normal (*n* = 7)	HAND (*n* = 15)		
		
		ANI (*n* = 7)	MND (*n* = 3)	HAD (*n* = 5)
**Demographics**				
Sex (f/m)	2/5	1/6	0/3	1/4
Years of age at death	55.9 ± 4.7	58.4 ± 5.6	52.7 ± 2.3	58.4 ± 5.6
Years of education	12.3 ± 2.7	14.1 ± 3.2	14 ± 3.5	11 ± 3
Race/ethnicity, *N* (%)				
White	5 (71.4%)	6 (85.7%)	3 (100%)	2 (40%)
Black	2 (28.6%)	1 (14.3%)	0 (0%)	0 (0%)
Asian	0 (0%)	0 (0%)	0 (0%)	1 (20%)
Other	0 (0%)	0 (0%)	0 (0%)	2 (40%)
**HIV disease characteristics**				
Duration of HIV diagnosis (years)	15.7 ± 6.8	14.1 ± 6.7	10.4 ± 0.2	13.6 ± 8.2
Antemortem plasma VL (log)	3.6 ± 3.9	5.2 ± 5.6	5.5 ± 5.6	5.1 ± 5.3
Antemortem CD4 count	272.9 ± 134.2	165.6 ± 150.3	87 ± 110.3	104 ± 125
Antemortem ART use, *N* (%)	6 (85.7%)	7 (100%)	3 (100%)	4 (80%)

### Complement component 3 protein is significantly upregulated in the frontal cortex of HIV brains with detectable beta amyloid plaques and in the frontal cortex from decedents with HIV-associated neurocognitive disorder

**FIGURE 1:**
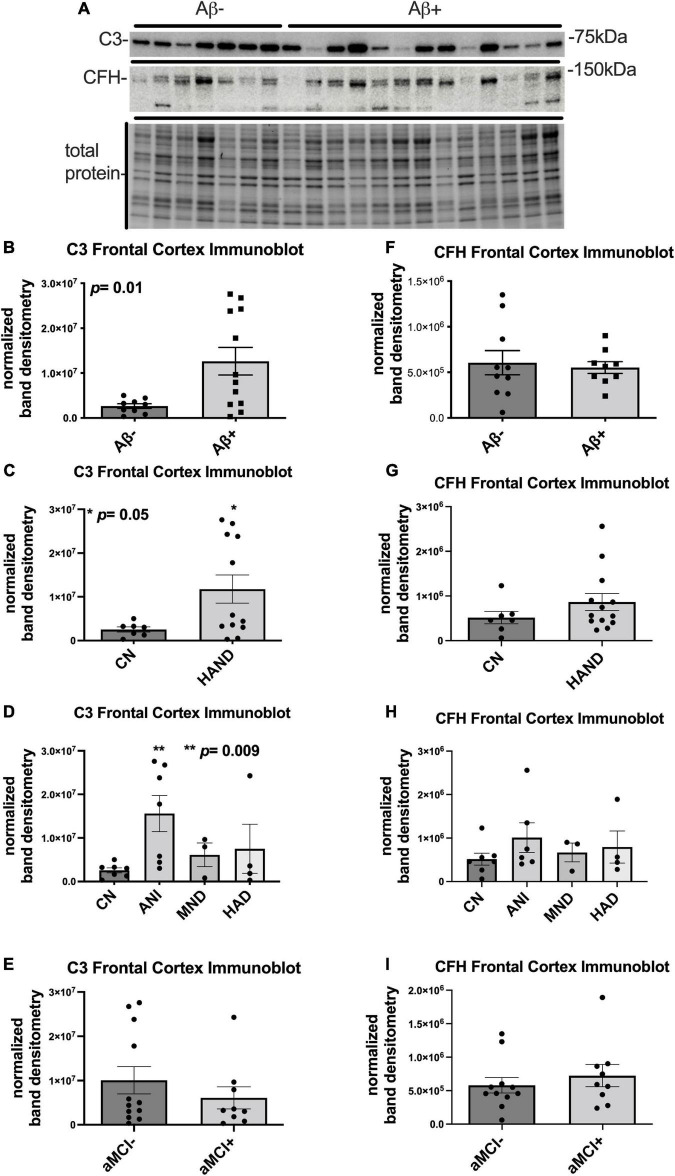
C3 protein is significantly upregulated in the frontal cortex of HIV brains with detectable Aβ plaques and in the frontal cortex from decedents with HAND. **(A)** Immunoblot for C3 and CFH and total protein transferred to the membrane using brain lysates from the frontal cortex from HIV brains that were determined to be negative or positive for Aβ plaques. **(B)** Quantification of C3 band intensity normalized to total protein transferred to the membrane stratified as negative or positive for Aβ plaques. **(C)** Quantification of C3 band intensity normalized to total protein and stratified by cognitive normal vs. HAND. **(D)** Quantification of C3 band intensity normalized to total protein and stratified by HAND sub-categories. **(E)** Quantification of C3 band intensity normalized to total protein and stratified by aMCI– vs. aMCI +. **(F)** Quantification of CFH band intensity normalized to total protein transferred to the membrane stratified as negative or positive for Aβ plaques. **(G)** Quantification of CFH band intensity normalized to total protein and stratified by cognitive normal vs. HAND. **(H)** Quantification of CFH band intensity normalized to total protein and stratified by HAND sub-categories. **(I)** Quantification of CFH band intensity normalized to total protein and stratified by aMCI– vs. aMCI +. Statistical significance was determined by an unpaired *t*-test (*p < 0.05, **p < 0.01).

To determine the expression levels of C3 and CFH protein in frontal cortices of HIV + donors with and without AD-related pathology and HAND, we performed immunoblot of lysates stratified by presence of Aβ plaques (Umlauf et al., 2019; Figure 1A). Bands corresponding to C3 and CFH proteins were detected at approximately 75 and 150 kDa, respectively (Figure 1A). A second band at approximately 100 kDa is apparent in the CFH blot; however, this is not consistent with the predicted molecular weight of CFH and, to our knowledge this band has not been reported in the literature. Future studies may be necessary to determine the biological relevance of the 105 kDa band. When normalized to total protein transferred to the membrane, the average intensity of the band corresponding to C3 showed a significant (∼5-fold) increase in the Aβ + group compared to the Aβ- group (Figure 1B). Comparing C3 band intensity in CN vs. HAND, C3 was significantly higher in the HAND group as a whole compared to the CN group [*F*_(1, 19)_ = 4.72, *p* = 0.04, Cohen’s *d* = 1.52; Figure 1C]. When stratified by HAND subgroups, the average intensity of the band corresponding to C3 was significantly higher (∼7-fold) in the ANI group compared to the CN group [*F*_(1, 12)_ = 9.76, *p* = 0.009; Figure 1D]. There was no significant difference in C3 band intensity in aMCI- vs. aMCI + groups [*F*_(1, 19)_ = 0.91, Cohen’s *d* = 0.43, *p* = 0.35; Figure 1E]. The average intensity of the band corresponding to CFH was not significantly different between the Aβ- and Aβ + groups (Figure 1F), the HAND and CN groups [*F*_(1, 18)_ = 0.54, *p* = 0.47; Figures 1G,H], or the aMCI- and aMCI + groups [*F*_(1, 18)_ = 0.69, Cohen’s *d* = 0.32, *p* = 0.42; Figure 1I]. We found no significant relationship between C3 or CFH levels in the FC and viral loads or CD4 + cell counts.

### Complement component 3 protein trends upward in the cerebellum of human immunodeficiency virus brains with detectable beta amyloid plaques

**FIGURE 2:**
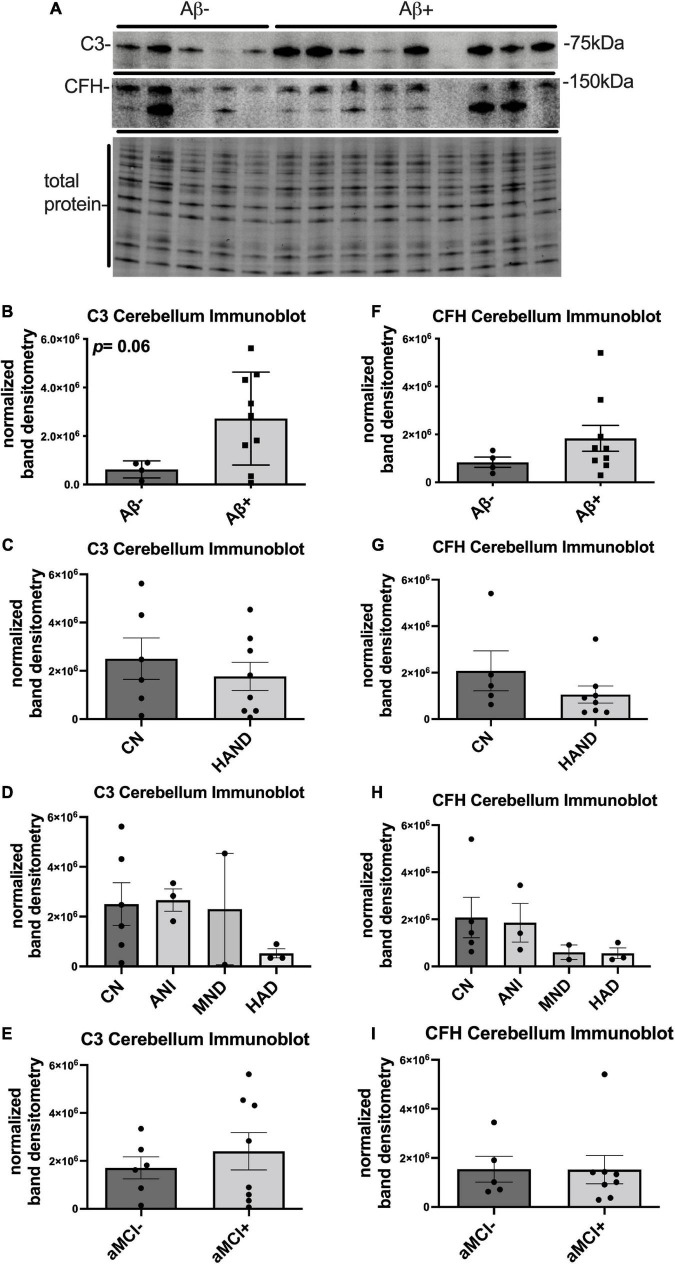
C3 protein trends upward in the cerebellum HIV brains with detectable Aβ plaques. **(A)** Immunoblot for C3 and CFH and total protein transferred to the membrane using brain lysates from the cerebellum from HIV brains that were determined to be negative or positive for Aβ plaques. **(B)** Quantification of C3 band intensity normalized to total protein transferred to the membrane stratified as negative or positive for Aβ plaques. **(C)** Quantification of C3 band intensity normalized to total protein and stratified by cognitive normal vs. HAND. **(D)** Quantification of C3 band intensity normalized to total protein and stratified by HAND sub-categories. **(E)** Quantification of C3 band intensity normalized to total protein and stratified by aMCI– vs. aMCI+. **(F)** Quantification of CFH band intensity normalized to total protein transferred to the membrane stratified as negative or positive for Aβ plaques. **(G)** Quantification of CFH band intensity normalized to total protein and stratified by cognitive normal vs. HAND. **(H)** Quantification of CFH band intensity normalized to total protein and stratified by HAND sub-categories. **(I)** Quantification of CFH band intensity normalized to total protein and stratified by aMCI– vs. aMCI+. Statistical significance was determined by an unpaired *t*-test.

To determine the expression levels of C3 and CFH protein in the cerebellum of HIV + donors with and without AD-related pathology and HAND, we performed immunoblot of lysates stratified by presence of Aβ plaques (Umlauf et al., 2019; Figure 2A). Bands corresponding to C3 and CFH proteins were detected at approximately 75 and 150 kDa, respectively (Figure 2A). When normalized to total protein transferred to the membrane, the average intensity of the band corresponding to C3 was ∼5-fold higher in Aβ + group compared to the Aβ- group (Figure 2B), though this difference did not reach significance. The average intensity of the band corresponding to C3 did not significantly differ in HAND compared to the CN group (Figure 2C) nor when stratified by HAND subgroups (Figure 2D). There was no significant difference in C3 band intensity in aMCI- vs. aMCI + groups (Figure 2E). The average intensity of the band corresponding to CFH was not significantly different between the Aβ- and Aβ + groups (Figure 2F), the HAND and CN groups (Figures 2G,H), or the aMCI- and aMCI + groups (Figure 2I). We found no significant relationship between C3 or CFH levels in the CB and viral loads or CD4 + cell counts.

### Complement component 3 protein expression is higher in the frontal cortex than the cerebellum and complement factor H protein expression is higher in the cerebellum than in the frontal cortex

**FIGURE 3:**
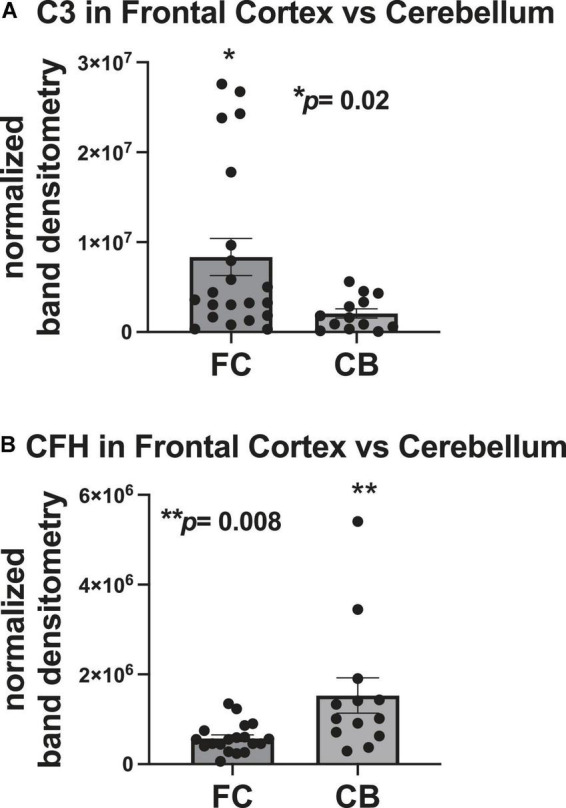
C3 protein expression is higher in the frontal cortex than the cerebellum and CFH protein expression is higher in the cerebellum than in the frontal cortex. **(A)** Quantification of C3 band intensity normalized to total protein and stratified by frontal cortex vs. cerebellum. **(B)** Quantification of CFH band intensity normalized to total protein transferred to the membrane stratified by frontal cortex vs. cerebellum. Statistical significance was determined by an unpaired *t*-test (*p < 0.05, **p < 0.01).

To determine differences in C3 and CFH expression in the FC and cerebellum, densitometry analyses quantities for each protein were compared between the two brain regions. The mean densitometry levels for C3 were significantly higher (∼4-fold) in the FC than in the cerebellum (Figure 3A). The mean densitometry levels for CFH were significantly higher (∼2.5-fold) in the cerebellum than in the FC (Figure 3B).

### Complement factor H protein levels relate to motor (frontal and cerebellar complement factor H) and executive function (frontal complement factor H only) performance

**FIGURE 4:**
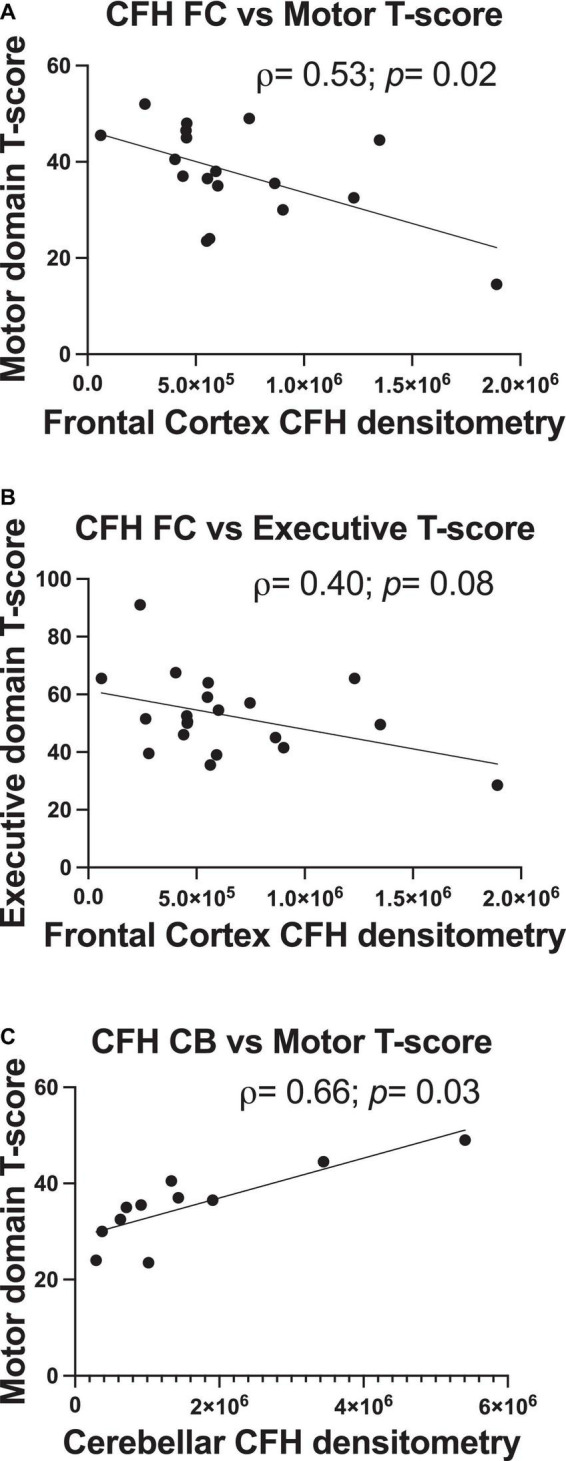
Frontal and cerebellar CFH protein levels relate to antemortem performance in specific cognitive domains. Quantification of frontal cortex CFH band intensity relates to motor domain **(A)** and executive function domain performance **(B)**. Quantification of cerebellum CFH band intensity relates to motor domain performance **(C)**. Statistical significance was determined by a Spearman’s rank-order correlation (ρ = Spearman’s rank correlation coefficient).

In correlational analyses, frontal and cerebellar C3 levels did not relate to any domain-specific T-scores (*p*> 0.05). There were moderate-sized correlations between higher frontal CFH protein levels and poorer motor domain scores (ρ = 0.54, *p* = 0.02) and poorer executive domain performance (ρ = –0.40, *p* = 0.08; Figures 4A,B), with the former a significant relationship and the latter a statistical trend. Lower cerebellar CFH protein levels significantly related to poorer motor domain T-score (ρ = 0.66, *p* = 0.03; Figure 4C). Frontal or cerebellar CFH levels did not relate to any other domain-specific T-score (*p* > 0.05).

## Discussion

The results of this study provide further evidence for the involvement of neuroinflammation and innate immune activation, with a specific emphasis on the complement system in the neuropathogenesis of HAND. This study also provides evidence for a role of the complement system at the nexus of HAND and AD neuropathogenesis. We identified increased C3 protein levels in FC tissues from decedents with HIV previously shown to have Aβ plaques in the cortex and in those diagnosed with HAND. Interestingly, and somewhat unexpectedly, C3 protein levels trended higher in the cerebellum of tissues with Aβ plaques in the cortex, despite having had fewer specimens available to analyze. Although there were no direct correlations with overall HAND status, altered levels of CFH protein in the FC were associated with deficiencies in executive and motor function performance. These findings are consistent with previous findings showing that alterations in the complement system may play a role in the neurodegenerative process in HAND and AD, highlighting the need to better understand the therapeutic potential of targeting this system.

C3 has long been suspected of being involved in the neurodegenerative process of HAND and AD. Our findings are consistent with a finding that C3 is positively associated with metabolic alterations in aged PWH (Bryant et al., 2016) and a significant role for complement in AD (Morgan, 2018). Elevated C3 levels in HAND and Aβ + brains are also consistent with studies showing that reactive astrocytes produce C3 (Liddelow et al., 2017; Nitkiewicz et al., 2017; Clarke et al., 2018; Vallee and Fields, 2022) and may contribute to metabolic deficiencies in neurons in HAND and AD (Jiang and Cadenas, 2014; Yin et al., 2016; Fields et al., 2019; Swinton et al., 2019). However, the relationship between C3 and AD neuropathogenesis is not clear. C3 deficiency promotes Aβ-associated neurotoxicity in animal models and enhanced levels of C3 in CSF are associated with MCI and AD (Wyss-Coray et al., 2002; Maier et al., 2008; Li et al., 2012; Lukiw and Alexandrov, 2012; Lukiw et al., 2012; Hoh Kam et al., 2013; Toledo et al., 2014; Hu et al., 2016; Zhang et al., 2016). C3 is also associated with the synaptic pruning process required for development and learning and memory formation (Schafer et al., 2012). This murky picture, when considering all available evidence, suggests that other factors are likely at play and interacting with the complement system to determine disease outcomes. Nevertheless, C3 consistently shows up in biomarker studies in association with HAND and AD neuropathogenesis, suggesting it may be useful as a therapeutic target or, when coupled with other biomarkers, a readout to determine therapeutic strategies and disease progression.

CFH plays a regulatory role in the complement pathway by dotting cells to be protected from C3 activity (Merle et al., 2015a,b). The elevated levels of CFH in the FC of PWH showing deficiencies in executive and motor function may represent a compensatory mechanism to reverse damage already done by C3 overactivation. On the other hand, CFH mutations and miRNA regulation have been shown to mute CFH function and be associated with disease (Li et al., 2012; Lukiw and Alexandrov, 2012; Lukiw et al., 2012; Zhang et al., 2016). In this case, higher levels may be negated by CFH dysfunction. However, further investigation into CFH sequences and miRNA expression in these brain tissues would be required to test this hypothesis. It is interesting that increases in CFH in the FC are not commensurate with the increases in C3 in the same brain tissues. This may indicate that more CFH in these tissues may protect cells from aberrant C3 overactivation. Moreover, the finding that C3 protein levels are higher in the FC than cerebellum while CFH protein levels are higher in the cerebellum than the FC adds a layer of complexity to interpreting the involvement of the complement system in HAND and AD. Triggering receptor on myeloid cells 2 (TREM2) is expressed on microglia and facilitates engulfment of synapses, dying cells, and Aβ (Kiialainen et al., 2005; Guerreiro et al., 2013). Increased levels of soluble TREM2 is associated with worse inflammation and neurodegenerative disease (Kiialainen et al., 2005; Benitez et al., 2013; Colonna and Wang, 2016; Henjum et al., 2016; Kobayashi et al., 2016). In light of recent reports of increased levels of soluble TREM2, by our group and others, may suggest pathogenic levels of synaptic pruning in PWH (Fields et al., 2018; Gisslen et al., 2019). It will be interesting to see if these differences are reflected in synaptic protein levels and synaptic health in the FC and cerebellum of these brain specimens. These findings suggest that along with C3, the role of CFH in neuropathogenesis of HIV in aging people deserves more attention.

The role of the cerebellum in HAND and AD has garnered little attention. However, recent studies suggest that the cerebellum is susceptible to Aβ deposition and toxicity (Hoxha et al., 2018). In brain specimens from humans and rodent models, Aβ deposition occurs in the region and is associated with synaptic damage (Sepulveda-Falla et al., 2011; Hoxha et al., 2012). While little is known about the cerebellum in HAND, our findings of C3 levels trending higher in the cerebellum in Aβ + brains are consistent with a role for this region in aging PWH. Similar to what we observed in the FC, the magnitude of change in CFH protein levels was not commensurate with C3 levels in the cerebellum. Moreover, the presence of motor dysfunction in many PWH may implicate a role for the cerebellum in neuropathogenesis of HIV. These findings in such a small cohort suggest more investigations are needed into the role of the cerebellum in PWH and neurological disorders.

In conclusion, our findings represent one of the first studies to investigate the role of the complement system in the context of AD-related neuropathogenesis in PWH. We also investigate for the first time the complement system in the cerebellum of HIV + brains in the context of AD-related neuropathogenesis. The discovery of strong associations in a cohort of limited size suggests more studies are necessary to determine the therapeutic and biomarker potential of C3 and CFH in aging PWH.

## Data availability statement

The raw data supporting the conclusions of this article will be made available by the authors, without undue reservation.

## Ethics statement

The studies involving human participants were reviewed and approved by the UCSD Institutional Review Board. The patients/participants provided their written informed consent to participate in this study.

## Author contributions

JF, ES, and DM designed the study, drafted, and edited the manuscript. MS, NS, and K-AV performed brain tissue analyses and edited the manuscript. All authors contributed to the article and approved the submitted version.
